# Supplementary figures and images for: Effects of the COVID-19 pandemic on life expectancy and premature mortality in the German federal states in 2020 and 2021
Source: PLoS One. 2023 Dec 21;18(12):e0295763. doi: 10.1371/journal.pone.0295763 (PMC10734971; doi:10.1371/journal.pone.0295763)

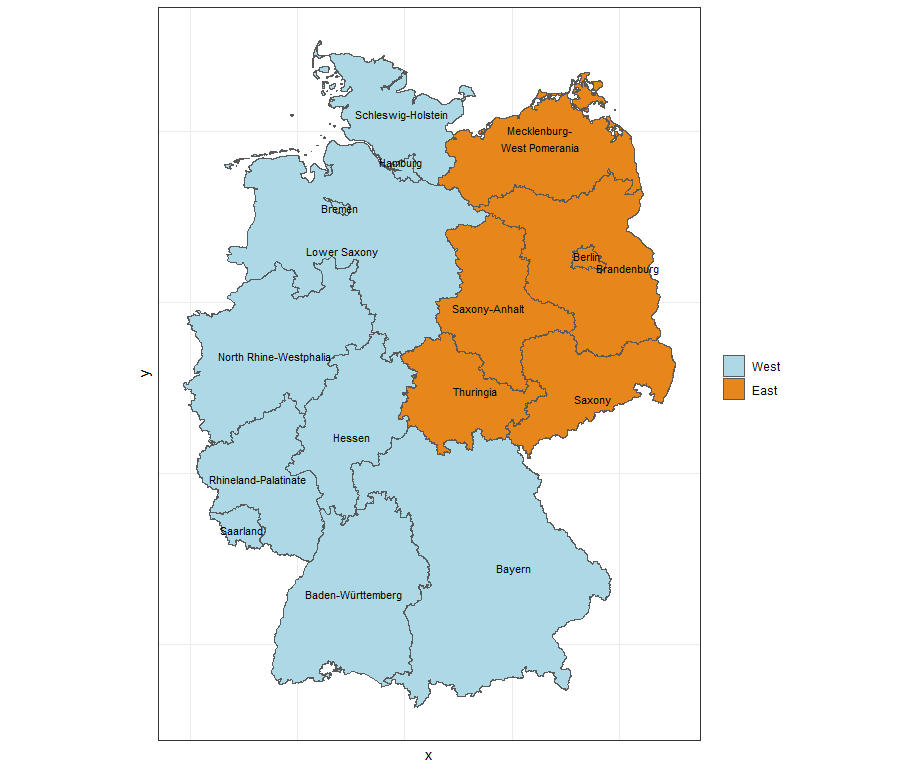

Supplement: S1 Fig — Reprinted from GADM shapefile under a CC BY license, with permission from GADM, original copyright 2018–2022. (TIF) [file pone.0295763.s001.tif]

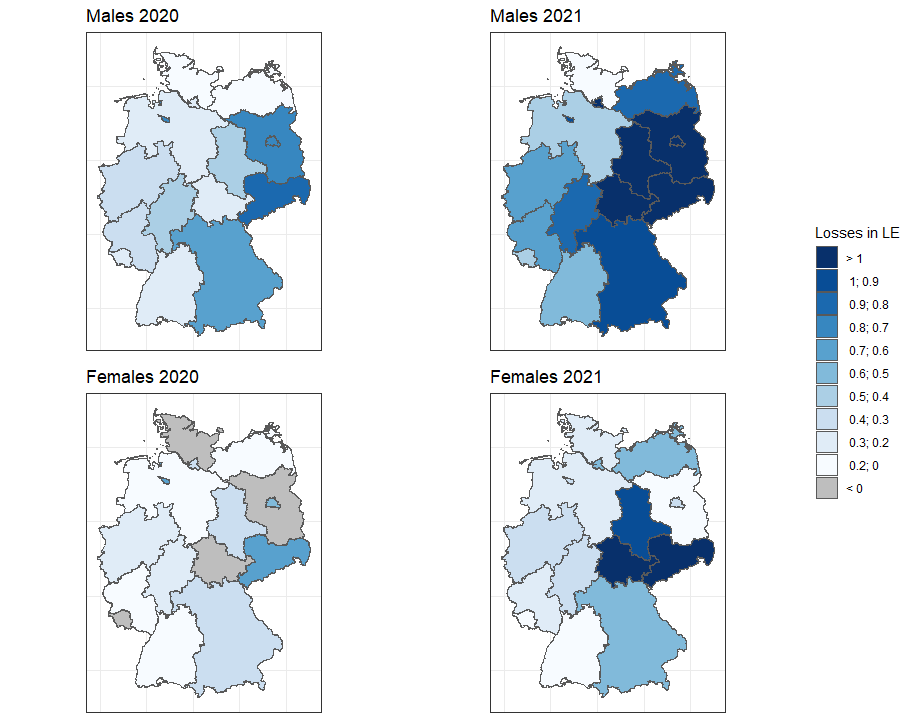

Supplement: S2 Fig — Reprinted from GADM shapefile under a CC BY license, with permission from GADM, original copyright 2018–2022. (TIF) [file pone.0295763.s002.tif]

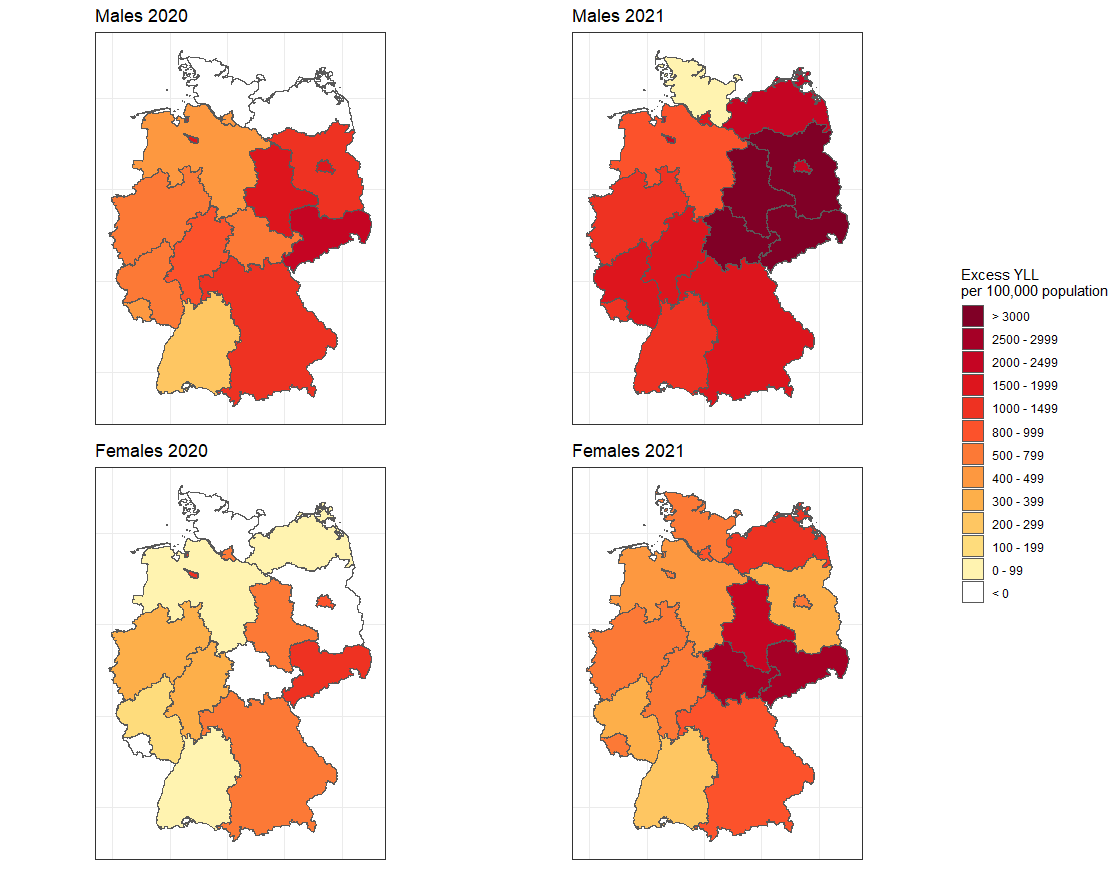

Supplement: S3 Fig — Reprinted from GADM shapefile under a CC BY license, with permission from GADM, original copyright 2018–2022. (TIF) [file pone.0295763.s003.tif]

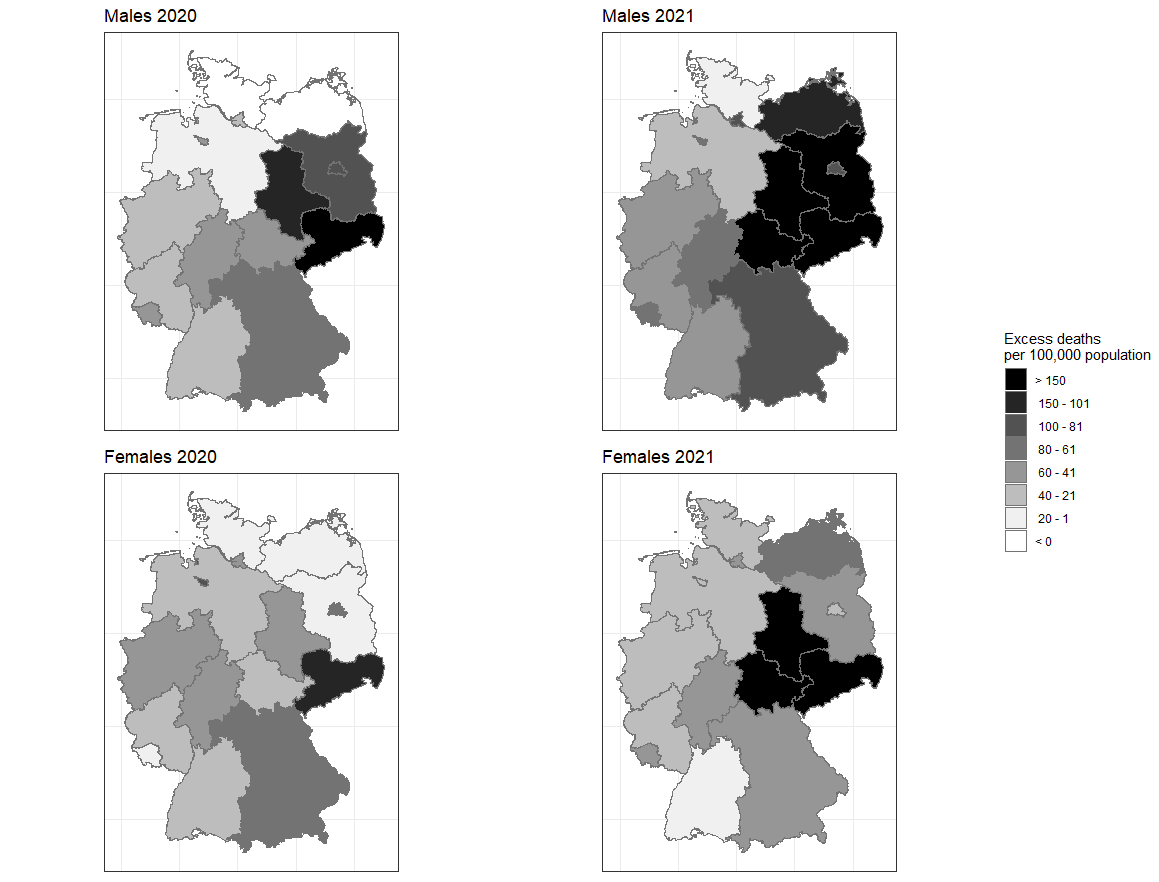

Supplement: S4 Fig — Reprinted from GADM shapefile under a CC BY license, with permission from GADM, original copyright 2018–2022. (TIF) [file pone.0295763.s004.tif]
