# Supplementary material for: Effects of the COVID-19 pandemic on life expectancy and premature mortality in the German federal states in 2020 and 2021
Source: PLoS One. 2023 Dec 21;18(12):e0295763. doi: 10.1371/journal.pone.0295763 (PMC10734971; doi:10.1371/journal.pone.0295763)
Supplement: S1 Table — (DOCX) [file pone.0295763.s005.docx]

**S1 Table. Population exposures of German federal states, Germany, and percentage (%) of population share**

|  |  | **2020** |  |  |  | **2021** |  |  |
| --- | --- | --- | --- | --- | --- | --- | --- | --- |
| **Federal State** | **Male** | **Female** | **Total** | **%** | **Male** | **Female** | **Total** | **%** |
| Baden-Württemberg | 5516860 | 5585559 | 11102419 | 13.3 | 5515442 | 5583601 | 11099043 | 13.4 |
| Bayern | 6510637 | 6622902 | 13133538 | 15.8 | 6509885 | 6623067 | 13132952 | 15.8 |
| Berlin | 1803077 | 1863426 | 3666503 | 4.4 | 1802328 | 1861934 | 3664262 | 4.4 |
| Brandenburg | 1246118 | 1280693 | 2526811 | 3.0 | 1243586 | 1278941 | 2522527 | 3.0 |
| Bremen | 336709 | 343957 | 680666 | 0.8 | 336107 | 343366 | 679474 | 0.8 |
| Hamburg | 905494 | 944411 | 1849905 | 2.2 | 907739 | 945834 | 1853573 | 2.2 |
| Hessen | 3107052 | 3184046 | 6291098 | 7.6 | 3105977 | 3180581 | 6286558 | 7.6 |
| Lower Saxony | 3949812 | 4049382 | 7999194 | 9.6 | 3945266 | 4045387 | 7990653 | 9.6 |
| Mecklenburg-West Pomerania | 793128 | 816477 | 1609605 | 1.9 | 790782 | 814488 | 1605269 | 1.9 |
| North Rhine-Westphalia | 8800934 | 9136413 | 17937347 | 21.6 | 8784917 | 9116925 | 17901842 | 21.6 |
| Rhineland-Palatinate | 2024680 | 2071786 | 4096466 | 4.9 | 2023133 | 2068927 | 4092061 | 4.9 |
| Saarland | 483745 | 501778 | 985523 | 1.2 | 481620 | 499243 | 980863 | 1.2 |
| Saxony | 2003122 | 2061866 | 4064989 | 4.9 | 1992040 | 2050559 | 4042598 | 4.9 |
| Saxony-Anhalt | 1076314 | 1111588 | 2187902 | 2.6 | 1068004 | 1103426 | 2171430 | 2.6 |
| Schleswig-Holstein | 1424394 | 1483230 | 2907625 | 3.5 | 1422578 | 1482185 | 2904764 | 3.5 |
| Thuringia | 1052696 | 1074307 | 2127003 | 2.6 | 1045366 | 1067235 | 2112601 | 2.5 |
| Germany | 41034772 | 42131821 | 83166594 |  | 40974770 | 42065699 | 83040470 |  |
