# Supplementary material for: Effects of the COVID-19 pandemic on life expectancy and premature mortality in the German federal states in 2020 and 2021
Source: PLoS One. 2023 Dec 21;18(12):e0295763. doi: 10.1371/journal.pone.0295763 (PMC10734971; doi:10.1371/journal.pone.0295763)
Supplement: S2 Table — (DOCX) [file pone.0295763.s006.docx]

**S2 Table. Losses in life expectancy at birth, 2020 and 2021**

|  | 2020… | | | | | |
| --- | --- | --- | --- | --- | --- | --- |
| **Federal State** | **Male (95% CI)** | | **Female (95% CI)** | | **Total (95% CI)** | |
| Baden-Württemberg | 0.203 | (0.188; 0.219) | 0.099 | (0.072; 0.127) | 0.158 | (0.138; 0.178) |
| Bayern | 0.633 | (0.616; 0.651) | 0.349 | (0.327; 0.371) | 0.508 | (0.489; 0.528) |
| Berlin | 0.794 | (0.771; 0.817) | 0.545 | (0.511; 0.583) | 0.694 | (0.665; 0.724) |
| Brandenburg | 0.771 | (0.748; 0.795) | 0.298 | (-0.405; -0.221) | 0.292 | (0.24; 0.341) |
| Bremen | 0.781 | (0.745; 0.818) | 0.638 | (0.59; 0.687) | 0.731 | (0.692; 0.772) |
| Hamburg | 0.257 | (0.233; 0.285) | 0.306 | (0.278; 0.333) | 0.287 | (0.26; 0.313) |
| Hessen | 0.495 | (0.467; 0.522) | 0.229 | (0.192; 0.263) | 0.379 | (0.35; 0.408) |
| Lower Saxony | 0.229 | (0.211; 0.247) | 0.018 | (-0.016; 0.053) | 0.131 | (0.105; 0.156) |
| Mecklenburg-West Pomerania | 0.121 | (0.09; 0.155) | 0.127 | (0.078; 0.175) | 0.124 | (0.085; 0.165) |
| North Rhine-Westphalia | 0.307 | (0.289; 0.328) | 0.207 | (0.177; 0.237) | 0.266 | (0.242; 0.291) |
| Rhineland-Palatinate | 0.361 | (0.341; 0.382) | 0.139 | (0.11; 0.17) | 0.262 | (0.237; 0.287) |
| Saarland | 0.225 | (0.18; 0.269) | -0.342 | (-0.395; -0.288) | -0.044 | (-0.09; 0.003) |
| Saxony | 0.866 | (0.836; 0.896) | 0.605 | (0.577; 0.632) | 0.768 | (0.739; 0.796) |
| Saxony-Anhalt | 0.456 | (0.342; 0.566) | 0.312 | (0.269; 0.352) | 0.383 | (0.309; 0.456) |
| Schleswig-Holstein | 0.003 | (-0.036; 0.038) | -0.051 | (-0.092; -0.009) | -0.024 | (-0.06; 0.013) |
| Thuringia | 0.254 | (0.227; 0.283) | -0.008 | (-0.039; 0.024) | 0.143 | (0.115; 0.171) |
| Germany | 0.41 | (0.391; 0.426) | 0.23 | (0.211; 0.257) | 0.32 | (0.31; 0.347) |

|  | 2021…… | | | | | |
| --- | --- | --- | --- | --- | --- | --- |
| **Federal State** | **Male** | | **Female** | | **Total** | |
| Baden-Württemberg | 0.539 | (0.516; 0.562) | 0.172 | (0.133; 0.21) | 0.374 | (0.345; 0.404) |
| Bayern | 0.927 | (0.903; 0.952) | 0.558 | (0.528; 0.589) | 0.764 | (0.737; 0.792) |
| Berlin | 1.055 | (1.021; 1.091) | 0.337 | (0.286; 0.39) | 0.733 | (0.692; 0.778) |
| Brandenburg | 1.484 | (1.45; 1.519) | 0.038 | (-0.223; 0.089) | 0.849 | (0.733; 0.908) |
| Bremen | 0.961 | (0.91; 1.015) | 0.348 | (0.276; 0.417) | 0.687 | (0.633; 0.745) |
| Hamburg | 1.079 | (1.042; 1.116) | 0.571 | (0.531; 0.613) | 0.854 | (0.817; 0.891) |
| Hessen | 0.868 | (0.83; 0.91) | 0.370 | (0.314; 0.421) | 0.645 | (0.605; 0.692) |
| Lower Saxony | 0.433 | (0.404; 0.461) | 0.236 | (0.185; 0.288) | 0.348 | (0.31; 0.385) |
| Mecklenburg-West Pomerania | 0.891 | (0.844; 0.938) | 0.570 | (0.498; 0.643) | 0.765 | (0.708; 0.821) |
| North Rhine-Westphalia | 0.648 | (0.62; 0.677) | 0.355 | (0.314; 0.398) | 0.519 | (0.486; 0.556) |
| Rhineland-Palatinate | 0.696 | (0.667; 0.727) | 0.259 | (0.213; 0.303) | 0.503 | (0.467; 0.538) |
| Saarland | 0.403 | (0.337; 0.469) | 0.106 | (0.023; 0.185) | 0.265 | (0.196; 0.333) |
| Saxony | 1.828 | (1.788; 1.87) | 1.173 | (1.135; 1.213) | 1.569 | (1.529; 1.611) |
| Saxony-Anhalt | 1.514 | (1.282; 1.693) | 0.991 | (0.928; 1.052) | 1.297 | (1.159; 1.41) |
| Schleswig-Holstein | 0.011 | (-0.043; 0.065) | 0.264 | (0.204; 0.325) | 0.129 | (0.073; 0.184) |
| Thuringia | 1.802 | (1.762; 1.841) | 1.224 | (1.181; 1.268) | 1.582 | (1.541; 1.623) |
| Germany | 0.60 | (0.578; 0.629) | 0.264 | (0.228; 0.297) | 0.44 | (0.414; 0.47) |
