# Supplementary material for: Effects of the COVID-19 pandemic on life expectancy and premature mortality in the German federal states in 2020 and 2021
Source: PLoS One. 2023 Dec 21;18(12):e0295763. doi: 10.1371/journal.pone.0295763 (PMC10734971; doi:10.1371/journal.pone.0295763)
Supplement: S3 Table — (DOCX) [file pone.0295763.s007.docx]

**S3 Table. Table with excess YLL and excess YLL per 100,000 population, by federal state and by sex, 2020 and 2021**

| 2020 | | | | | | |
| --- | --- | --- | --- | --- | --- | --- |
|  | ***Excess YLL*** | | | ***Excess YLL per 100,000 population*** | | |
| Federal state | **Male** | **Female** | **Total** | **Male** | **Female** | **Total** |
| Baden-Württemberg | 16434.8 | 1091.8 | 17526.6 | 297.9 | 19.5 | 317.4482 |
| Bayern | 78679.1 | 37544.8 | 116223.9 | 1208.5 | 566.9 | 1775.364 |
| Berlin | 27157.2 | 15130.0 | 42287.2 | 1506.2 | 811.9 | 2318.104 |
| Brandenburg | 18518.1 | -6813.3 | 11704.8 | 1486.1 | -532.0 | 954.0621 |
| Bremen | 5641.0 | 3896.8 | 9537.8 | 1675.3 | 1132.9 | 2808.266 |
| Hamburg | 4474.7 | 4879.6 | 9354.3 | 494.2 | 516.7 | 1010.854 |
| Hessen | 28733.6 | 10006.3 | 38739.9 | 924.8 | 314.3 | 1239.05 |
| Lower Saxony | 16744.0 | 1554.6 | 18298.6 | 423.9 | 38.4 | 462.31 |
| Mecklenburg-West Pomerania | -1387.8 | 585.4 | -802.4 | -175.0 | 71.7 | -103.28 |
| North Rhine-Westphalia | 52388.3 | 28943.4 | 81331.7 | 595.3 | 316.8 | 912.0501 |
| Rhineland-Palatinate | 12241.5 | 4027.2 | 16268.7 | 604.6 | 194.4 | 798.9971 |
| Saarland | 2385.3 | -1992.2 | 393.1 | 493.1 | -397.0 | 96.0622 |
| Saxony | 46739.1 | 30823.7 | 77562.8 | 2333.3 | 1494.9 | 3828.255 |
| Saxony-Anhalt | 16614.9 | 5824.7 | 22439.6 | 1543.7 | 524.0 | 2067.683 |
| Schleswig-Holstein | -3326.0 | -644.4 | -3970.4 | -233.5 | -43.4 | -276.949 |
| Thuringia | 6491.3 | -839.9 | 5651.4 | 616.6 | -78.2 | 538.4551 |
| Germany | 328529.1 | 134018.5 | 462547.6 | 800.6 | 318.1 | 1118.7 |

| 2021 | | | | | | | | |
| --- | --- | --- | --- | --- | --- | --- | --- | --- |
|  | ***Excess YLL*** | | | | ***Excess YLL per 100,000 population*** | | | |
| Federal state | **Male** | | **Female** | **Total** | **Male** | **Female** | | **Total** |
| Baden-Württemberg | 55959.6 | | 11368.9 | 9862.2 | 1014.6 | 203.6 | 665.4 | |
| Bayern | 120538.7 | | 63612.1 | 26612.6 | 1851.6 | 960.5 | 2892.4 | |
| Berlin | 37234.7 | | 10645.5 | 54593.7 | 2065.9 | 571.7 | 1373.1 | |
| Brandenburg | 45260.9 | | 4873.3 | 8996.6 | 3639.6 | 381.0 | 2662.6 | |
| Bremen | 6744.9 | | 2251.7 | 181585.3 | 2006.8 | 655.8 | 2042.1 | |
| Hamburg | 17751.9 | | 8860.7 | 76222.6 | 1955.6 | 936.8 | 2438.1 | |
| Hessen | 55066.3 | | 21156.3 | 38400.5 | 1772.9 | 665.2 | 1889.9 | |
| Lower Saxony | 37576.0 | | 17017.7 | 67328.5 | 952.4 | 420.7 | 1218.2 | |
| Mecklenburg-West Pomerania | 19117.8 | | 10090.1 | 184150.8 | 2417.6 | 1238.8 | 2812.1 | |
| North Rhine-Westphalia | 121541.9 | | 60043.4 | 8392.8 | 1383.5 | 658.6 | 1721.7 | |
| Rhineland-Palatinate | 30950.1 | | 7450.4 | 47880.2 | 1529.8 | 360.1 | 2637.6 | |
| Saarland | 5541.4 | | 2851.4 | 50134.2 | 1150.6 | 571.1 | 4020.6 | |
| Saxony | 90121.4 | | 51732.6 | 29207.9 | 4524.1 | 2522.9 | 3656.4 | |
| Saxony-Anhalt | 51727.8 | | 25822.4 | 141854 | 4843.4 | 2340.2 | 7047 | |
| Schleswig-Holstein | 10.3 | | 9851.9 | 77550.2 | 0.7 | 664.7 | 7183.6 | |
| Thuringia | 52792.7 | | 30677.8 | 83470.5 | 5050.2 | 2874.5 | 7924.7 | |
| Germany | 747936.4 | 338306.2 | | 1086242.6 | 1825.358 | 804.2329 | 2629.591 | |
