# Supplementary material for: Effects of the COVID-19 pandemic on life expectancy and premature mortality in the German federal states in 2020 and 2021
Source: PLoS One. 2023 Dec 21;18(12):e0295763. doi: 10.1371/journal.pone.0295763 (PMC10734971; doi:10.1371/journal.pone.0295763)
Supplement: S4 Table — (DOCX) [file pone.0295763.s008.docx]

**S4 Table. Table with excess deaths and excess deaths per 100,000 population, by federal state and by sex, 2020 and 2021**

| 2020 | | | | | | |
| --- | --- | --- | --- | --- | --- | --- |
|  | ***Excess deaths*** | | | ***Excess deaths per 100,000 population*** | | |
| Federal state | **Male** | **Female** | **Total** | **Male** | **Female** | **Total** |
| Baden-Württemberg | 1291.1 | 886.1 | 2177.2 | 23.4 | 15.9 | 39.3 |
| Bayern | 4145.9 | 2721.0 | 6866.9 | 63.7 | 41.1 | 104.8 |
| Berlin | 1458.4 | 1032.0 | 2490.4 | 80.9 | 55.4 | 136.3 |
| Brandenburg | 997.2 | -141.4 | 855.8 | 80.0 | -11.0 | 69 |
| Bremen | 200.9 | 222.8 | 423.7 | 59.7 | 64.8 | 124.5 |
| Hamburg | 357.0 | 372.2 | 729.2 | 39.4 | 39.4 | 78.8 |
| Hessen | 1448.5 | 1205.0 | 2653.5 | 46.6 | 37.8 | 84.4 |
| Lower Saxony | 414.8 | 61.6 | 476.4 | 10.5 | 1.5 | 12 |
| Mecklenburg-West Pomerania | -246.7 | -168.2 | -414.9 | -31.1 | -20.6 | -51.7 |
| North Rhine-Westphalia | 2754.7 | 2163.9 | 4918.6 | 31.3 | 23.7 | 55 |
| Rhineland-Palatinate | 443.7 | 177.7 | 621.4 | 21.9 | 8.6 | 30.5 |
| Saarland | 240.7 | -74.4 | 166.3 | 49.8 | -14.8 | 35 |
| Saxony | 3115.9 | 2877.7 | 5993.6 | 155.6 | 139.6 | 295.2 |
| Saxony-Anhalt | 1549.1 | 380.5 | 1929.6 | 143.9 | 34.2 | 178.1 |
| Schleswig-Holstein | -330.7 | -97.3 | -428 | -23.2 | -6.6 | -29.8 |
| Thuringia | 445.1 | 86.2 | 531.3 | 42.3 | 8.0 | 50.3 |
| Germany | 18285.6 | 11705.4 | 29991 | 44.6 | 27.8 | 72.3 |

| 2021 | | | | | | | | | |
| --- | --- | --- | --- | --- | --- | --- | --- | --- | --- |
|  |  | ***Excess deaths*** | | | | ***Excess deaths per 100,000 population*** | | | |
| Federal state | **Male** | | **Female** | **Total** | | **Male** | | **Female** | **Total** |
| Baden-Württemberg | 2331.0 | | 942.0 | | 554.7 | 42.3 | 16.9 | | 37.5 |
| Bayern | 5656.6 | | 3637.3 | | 1296.8 | 86.9 | 54.9 | | 140.6 |
| Berlin | 1588.4 | | 536.7 | | 2038.9 | 88.1 | 28.8 | | 51.2 |
| Brandenburg | 2274.6 | | 582.3 | | 351.7 | 182.9 | 45.5 | | 104.1 |
| Bremen | 261.7 | | 90.0 | | 8332.5 | 77.9 | 26.2 | | 93.4 |
| Hamburg | 785.0 | | 511.8 | | 3994.6 | 86.5 | 54.1 | | 127.3 |
| Hessen | 2349.0 | | 1645.6 | | 1546.2 | 75.6 | 51.7 | | 75.9 |
| Lower Saxony | 1166.7 | | 872.2 | | 3273 | 29.6 | 21.6 | | 59.2 |
| Mecklenburg-West Pomerania | 906.3 | | 587.5 | | 9293.9 | 114.6 | 72.1 | | 141.8 |
| North Rhine-Westphalia | 4887.1 | | 3445.4 | | 577.3 | 55.6 | 37.8 | | 118.3 |
| Rhineland-Palatinate | 1060.5 | | 485.7 | | 2125.1 | 52.4 | 23.5 | | 116.9 |
| Saarland | 364.7 | | 212.6 | | 2856.9 | 75.7 | 42.6 | | 228.4 |
| Saxony | 4440.3 | | 3399.5 | | 1493.8 | 222.9 | 165.8 | | 186.7 |
| Saxony-Anhalt | 3337.7 | | 1666.3 | | 7839.8 | 312.5 | 151.0 | | 388.7 |
| Schleswig-Holstein | 31.7 | | 523.0 | | 53394.1 | 2.2 | 35.3 | | 463.5 |
| Thuringia | 2692.4 | | 2117.0 | | 54909.7 | 257.6 | 198.4 | | 456 |
| Germany | 34133.6 | | 21254.9 | | 153879 | 83.3 | 50.5 | | 133.8 |
