# Supplementary material for: Effects of the COVID-19 pandemic on life expectancy and premature mortality in the German federal states in 2020 and 2021
Source: PLoS One. 2023 Dec 21;18(12):e0295763. doi: 10.1371/journal.pone.0295763 (PMC10734971; doi:10.1371/journal.pone.0295763)
Supplement: Attachment — Submitted filename: Rebuttal letter_20231014.docx [file pone.0295763.s009.docx]

**Manuscript PONE-D-23-10425**

**Response to Reviewers**

Dear Academic Editor, thank you for giving us the opportunity to submit a revised draft of the manuscript “Effects of the COVID-19 pandemic on life expectancy and premature mortality in the German federal states in 2020 and 2021” for publication in PLOS ONE. We appreciate the time and effort that you and the reviewers dedicated to providing feedback on our manuscript.

We have incorporated most of the suggestions made by the reviewers, throughout the whole manuscript. Those changes are highlighted within the manuscript. Please see below the questions/comments raised by the reviewers (in italics) and our responses. All page numbers refer to the revised manuscript file with tracked changes.

*Reviewer #1:*

*Thank you for conducting such a study, there are several things needed to be further clarified*

Author response: Thank you. Please see the below revision to the manuscript according to your comments.

*1. In Data and Method section, state clearly the study design, the inclusion and exclusion criteria, the source of data, the time frame of the study, and the ethical permission to conduct this study*

Authors response: This is an ecological study. Thus, we are using population-level mortality data provided by the German statistical office. There is no need for ethical permission. The Data and Method section has been updated to clarify this, and we also added a sentence on our study design and the data source in our abstract. Thank you for the advice.

Pages 5-6: This study aimed to assess the mortality impact of COVID-19 in Germany and its regions at the level of the 16 federal states (S1 Fig and S1 Table) by sex in 2020 and 2021, using the excess mortality method [4]. This is the most robust and reliable approach to quantifying the mortality burden due to the pandemic [21]. The excess mortality was estimated as the difference between the expected and observed mortality. For this purpose, we adopted an ecological study design, using population-level mortality data by federal state, age, and sex, for the years 2005-2021 obtained from the German Statistical Office. These data are available for everybody upon request and are part of a standard statistical report. No ethical approval is required.

*2. In the discussion section: write about the limitations of the study.*

Authors response: Thank you for the suggestion. We included a limitation section in the discussion.

Page 16: Limitations of the study

Some limitations of the study must be considered. First of all, when applying the excess mortality method to small numbers the obtained results are less robust and should be interpreted with caution. In our study, this applies particularly to the federal state of Bremen, with a population size of around 600 thousand inhabitants. Second, the method and fitting period used for the computation of the expected level of mortality – to compare the observed mortality level to it- should be carefully chosen to avoid an under- or overestimation of the estimates of the mortality impact of COVID-19. In our study, we avoided bias as much as possible by using a long series (15 years) of historical data and by employing the benchmark Lee-Carter mortality projection method. Nevertheless, using a different projection method and a different fitting period may lead to slightly different results. Third, when COVID-19 deaths are not many it is harder to statistically isolate the mortality impact of the pandemic, independent of the method. Still, however, the first and third limitations are both (partly) inherent to subnational level studies and should not prevent from undertaking these results because of the additional insights they still bring.

Reviewer #2:

*Researchers must follow the instructions of the journal*

Authors response: The manuscript has been revised to meet the PLOS ONE style requirements.

*Introduction
The impacts of COVID_19 have also been captured in numerous studies that are appearing elsewhere, or that are currently under review. Please consider revising your manuscript to speak directly to the global literature - and debates about covid-19 and mortality. That would help you to better situate the original contribution of this research.
The introduction should briefly place the study in a broad context and highlight why it is important. It should define the purpose of the work and its significance. The authors should review the current state of the research and document key publications. Please highlight controversial and diverging hypotheses when necessary. Finally, briefly mention the main aim of the work and highlight the principal conclusions.*

Authors response: Thank you for the comment. The paper is focused on spatial inequality in COVID-19 burden at subnational level. Due to limited data availability, there is lack of publications on this topic. Nevertheless, we agree that providing more info on the global context helps to better situate the manuscript’s contribution to the existing COVID-19 literature. Thus, we revised the introduction in response to the comments. In particular, we added several key publications on the broad topic of COVID-19 and mortality to the Introduction section.

Page 3: “At the end of 2021, more than 5 million deaths attributable to COVID-19 have been estimated around the world [1].

Many studies have been conducted to understand the magnitude of the impact of the COVID-19 pandemic on mortality at the national level [1-7]. These national estimates, however, hide an uneven impact of the pandemic across subnational populations [8]. Indeed, previous studies have shown important inequalities in the mortality impact of COVID-19 by region [8-12], sex [13-15], socio-economic status [16-18], and race [17-19], and generally, higher case-fatality rates have been reported among older age groups [14, 20]. Still, however, more work is needed to obtain a detailed picture of the mortality impact of the COVID-19 epidemic.”

Moreover, in the final paragraph of the introduction [page 5], we have clarified the objective of the study and included the overall findings:

“Overall, we found an unequal distribution of the mortality impact of COVID-19 at the subnational level in Germany, particularly among the male population, which increased over time.”

*Methodology*

*The methodology section is disorganized and not carefully written, the Materials and Methods should be described with sufficient details to allow others to replicate and build on the published results*

Authors response: We have revised the data and methods section to allow a better understanding of the underlying data and the methodologies used to obtain the presented results [pages 5-7]. In particular, we provide a brief overview of the methodology and data:

Page 5-6: This study aims to assess the mortality impact of COVID-19 in Germany and its regions at the level of the 16 federal states (S1 Fig and S1 Table) by sex in 2020 and 2021, using the excess mortality method [4]. This is the most robust and reliable approach to quantifying the mortality burden due to the pandemic [21]. The excess mortality was estimated as the difference between the expected and observed mortality. For this purpose, we adopted an ecological study design, using population-level mortality data by federal state, age, and sex, for the years 2005-2021 obtained from the German Statistical Office. These data are available for everybody upon request and are part of a standard statistical report. No ethical approval is required.”

We restructured the provided information, and – where necessary - added details to allow others to replicate and build on the published results. In particular, we have divided the Data & Methods section into subsections (“Observed mortality in 2020 and 2021”, “Expected mortality in 2020 and 2021”, “Mortality indicators”, and “Confidence intervals”), reorganized all 4 subsections to make the results replicable and added the YLL formula.

Moreover, the minimal dataset and the Rscript used for the computations are available on an OSF repository (DOI 10.17605/OSF.IO/V2MRG).

*Results*

*The symbols are written incorrectly as the life expectancy. e0*

Authors response: Thank you for pointing this out. The life expectancy symbol has now been changed into LE (and e0_loss into LE_ loss) throughout the whole manuscript, as also used in other similar papers (see Schöley et al. 2022 <https://www.nature.com/articles/s41562-022-01450-3>). Moreover, the previous symbol for excess Years of Life Lost has been changed from Excess_YLL to Excess YLL for better readability of the manuscript.

*discussion
Authors should discuss the results and how they can be interpreted from the perspective of previous studies and of the working hypotheses. The findings and their implications should be discussed in the broadest context possible. Future research directions may also be highlighted.*

Authors response: As suggested by the reviewer, we have reviewed the discussion part, dividing it into: “Main findings”, “Limitations of the study”, and “Conclusions”. Furthermore, we have added a comparison of our results with previous studies on the mortality impact of COVID-19 at the subnational level (paragraph 2 of the Discussion). We now placed our results in the wider context of the effect of COVID on growing sub-national inequalities and lastly, we added future research directions in line with this:

Page 17: “Further research should be carried out not only to understand the potential long-term impact of the COVID-19 pandemic on long-term longevity trends, but also on their effects on widening health and mortality inequalities within countries.”
