# Supplementary material for: Effects of the COVID-19 pandemic on life expectancy and premature mortality in the German federal states in 2020 and 2021
Source: PLoS One. 2023 Dec 21;18(12):e0295763. doi: 10.1371/journal.pone.0295763 (PMC10734971; doi:10.1371/journal.pone.0295763)
Supplement: Attachment — Submitted filename: Rebuttal letter_20231120.docx [file pone.0295763.s010.docx]

**Manuscript PONE-D-23-10425R1**

**Response to Reviewers**

Dear Dr. Dávila-Cervantes,

Thank you for giving us the opportunity to submit a revised draft of the manuscript “Effects of the COVID-19 pandemic on life expectancy and premature mortality in the German federal states in 2020 and 2021”, for reconsideration for publication PLOS ONE. We would like to thank you and the reviewers for suggesting minor, although constructive, revisions to our manuscript. The recommendations and advice have helped us enhance the manuscript's quality. In particular, in accordance with your request, the reference list has been modified to ensure that it is complete and correct, visible in tracked changes of the manuscript.

In accordance with Reviewer 3#’s comments, we have modified the conclusions and limitations of the study adding two more points. Moreover, we have added the reference to the data and scripts OSF repository in the Data and Methods section (page 5). Our revisions to the text are highlighted within the manuscript. Our point-by-point responses to the reviewers’ comments are shown below after the questions/comments raised by the reviewers (in italics). All page numbers refer to the revised manuscript file with tracked changes.

Reviewer #1:

*1. All required data and related information are included in the manuscript.
Thank you to correcting the manuscript as required.*

Author response: Thank you for your suggestions and the time spent on the revision of the manuscript.

Reviewer #3:

*1.I have very minor comments to the authors since this seems to be a revised version already. Some sentenced read oddly and would argue there are two additional limitations:
They could also describe limitations of the indicators: LE referring to a synthetic cohort, YLL being affected by population structure and death counts, SLE may pertain to a fictitious population and not Germany, and while they argue that their methodology is more accurate than others they do not present evidence of this. For example, how do results compare with methods used by Shoeley or Nepomuceno et al. ?*

Author response: Thank you for your comments. The manuscript has been revised according to your suggestions. Particularly:

Page 14: “First, although we used standard and commonly used mortality measures, they have their own limitations. Period life expectancy at birth (LE), as a summary measure, refers to a synthetic cohort and does not reflect a real mortality experience of any cohort; the premature mortality measure, years of life lost (YLL), depends entirely on the population structure and the death counts; and the WHO standardised life expectancy (SLE) used for the calculation of YLL refers to a fictitious population and is used in the analysis merely to allow the comparison of our results to the existing literature.”

Secondly, the sentence “However, our findings are in line with previous research, using different methods [Scholey 2021, Nepomuceno et al. 2022] and address robustness, bias and baseline approach issues.” [page 12] has been added to frame our findings and methodology in the context of validation and justification of the methods used in the analyses.

*2.here are some minor comments: Abstract: ‘Therefore, assess geographical… therein’ This sentence does not make sense, is lacking a purpose. Replace COVID-19 epidemic with COVID-19 pandemic. The sentence ‘Most of these research experienced methodological issues..’ reads oddly, perhaps rephrasing to ‘Mos to these research presents methodological limitations…
The phrase ‘advanced methodology’ is inaccurate. I would argue that the Lee-Carter method is standard and well known method.*

Author response: We have modified the manuscript according to your suggestions.
